# Influences of arbuscular mycorrhizal fungi on *Diospyros lotus* seedlings under salinity stress

**DOI:** 10.3389/fpls.2025.1595144

**Published:** 2025-07-23

**Authors:** Meral Incesu, Berken Cimen, Bilge Yilmaz, Turgut Yesiloglu, Ibrahim Ortas

**Affiliations:** ^1^ Department of Horticulture, Faculty of Agriculture, Cukurova University, Adana, Türkiye; ^2^ Department of Soil Science, Faculty of Agriculture, Cukurova University, Adana, Türkiye

**Keywords:** *D. lotus* plant growth, salt stress, plant nutrients, arbuscular mycorrhizal fungi, mycorrhizae dependency

## Abstract

Soil salinization, intensified by climate change, poses a growing threat to agricultural sustainability across the Mediterranean basin. As salinity levels rise in Mediterranean soils, the cultivation of salt-sensitive crops like persimmons is becoming increasingly vulnerable. This study investigated the effects of different arbuscular mycorrhizal fungi (AMF) species—*Glomus clarium* and *Claroideoglomus etunicatum—*on *Diospyros lotus* seedlings under varying salinity levels (0, 50, 100, and 150 mM NaCl). Seeds of *D. lotus* L. were used as a plant material, and the pot experiment was carried out under greenhouse conditions. Plant dry weight, chlorophyll, *Fv*/*Fm*, root colonization, and leaf and root mineral concentrations were investigated. Statistically, root colonization varied significantly with both mycorrhizal species and salinity levels, with *C. etunicatum* showing higher colonization rates than *G. clarium* across all treatments. Under saline conditions, both AMF species-inoculated plants exhibited significantly higher fresh and dry weights, chlorophyll content (SPAD), and photosystem II efficiency (*Fv*′/*Fm*′), and reduced symptom scores. *C. etunicatum* demonstrated superior tolerance to salinity, maintaining higher biomass and chlorophyll fluorescence at elevated salt concentrations. Mycorrhizal dependency values exceeded 70% under salinity, reflecting the critical role of AMF in enhancing stress resilience. It was determined that *D. lotus* seedlings are dependent on mycorrhiza and cannot grow in conditions without mycorrhiza inoculation. Mineral nutrient analysis revealed increased concentration of Ca, K, P, Mg, Fe, Mn, and Zn, and moderated Na and Cl accumulation in AMF-inoculated seedlings, with *G. clarium* particularly effective at limiting sodium translocation. These findings demonstrate that AMF inoculation, particularly with *C. etunicatum*, can effectively mitigate salinity-induced damage and improve nutrient balance, growth, and physiological performance in *D. lotus*. The results highlight the potential of mycorrhizal inoculation for sustainable cultivation in saline soil conditions.

## 1 Introduction

Persimmon cultivation in Turkey is increasing day by day. To date, persimmon production in Turkey has reached 60.661 tons (TUIK, 2024). Most of the production takes place in the Mediterranean region, and persimmon growth is becoming especially widespread in coastal areas.

However, orchards located near the sea areas are exposed to salt stress, and it is predicted that this stress will intensify further due to climate change. Salinity is considered one of the most harmful abiotic stresses for plant life cycles, and it is estimated that salt stress accounts for approximately 20% of potential crop yield loss (Pandit et al., 2024). Salinity stress adversely affects plant physiology through three primary mechanisms: (1) osmotic stress leading to cellular dehydration, (2) ion toxicity from excessive Na^+^ and Cl^-^ accumulation, and (3) oxidative damage caused by reactive oxygen species (ROS) (Incesu et al., 2014; Boorboori and Lackoova, 2025).

Three rootstocks are used in persimmon cultivation. Kaki is a rootstock widely used all over the world, but it is not used in the Mediterranean basin due to its low resistance to calcareous soils. The lotus rootstock tolerates calcareous soils. Although lotus is a suitable rootstock for calcareous soil, it is sensitive to salinity stress. It is predicted that there will be problems with the use of lotus in both calcareous and salinity-threatened soils of the Mediterranean region. Although the *D. virginiana* rootstock is superior to the other two rootstocks in terms of tolerance to both calcareous and saline soils, it is not desired to be used because it produces very large trees (LaRue et al., 1982; Bellini, 2002; Llácer et al., 2008; Gil-Muñoz et al., 2018; Izhaki et al., 2018; Gil-Muñoz et al., 2020).

It is predicted that due to climate change, rainfall will decrease in the Mediterranean basin, leading to the risk of drought, and the reduction in precipitation will cause salt stress in soils (Tramblay et al., 2020). The best solution against salt stress is the use of tolerant rootstocks (Forner-Giner and Ancillo, 2013). As salinity levels rise in Mediterranean soils, the cultivation of salt-sensitive crops like persimmons is becoming increasingly vulnerable. However, in cases where tolerant rootstocks are unavailable or the salt tolerance of the existing rootstock is insufficient, this can be improved using mycorrhiza. Introducing beneficial soil microorganisms to plants is an effective approach that not only enhances their resistance to salt stress but also boosts their overall productivity (Boorboori and Lackoova, 2025).

Arbuscular mycorrhizal fungi (AMF), classified within the order Glomales, function as crucial plant growth modulators that effectively mitigate salt stress-induced damage in host plants (Hameed et al., 2014). As the dominant symbiotic fungi in agricultural ecosystems, AMF form mutualistic associations with the root systems of approximately 90% of terrestrial plant species (Smith and Read, 2010). Research demonstrates that AMF enhance plant salt tolerance through five key physiological mechanisms: (1) enhanced nutrient uptake coupled with ionic balance regulation, (2) improved water absorption and osmotic adjustment, (3) activation of antioxidant defenses against ROS, (4) protection of photosynthetic machinery with concomitant efficiency gains, and (5) phytohormonal modulation to sustain growth under saline conditions (Evelin et al., 2019; Boorboori and Lackoova, 2025). There have been many studies showing that tolerance to salt stress can be increased with the use of mycorrhizae (Hajiboland, 2013; Evelin et al., 2019). It has been reported that mycorrhizal symbiosis increases the tolerance of many horticulture plants against salt stress such as eggplant (Mohammad and Mittra, 2013), zucchini (Colla et al., 2008), pomegranate (Yarahmadi Arab et al., 2018), tomato (Başak et al., 2011; Öztekin et al., 2013);, cucumber (Haghighi et al., 2017), pepper (Kaya et al., 2009; Beltrano et al., 2013; Altunlu, 2020; Basak et al., 2019), and citrus (Khalil et al., 2011; Rodríguez-Morán et al., 2015: Satir et al., 2016).

Turkey is located in the Mediterranean basin, and persimmon cultivation is primarily carried out in the Mediterranean region. In Turkey, kaki rootstock is predominantly used in persimmon cultivation, leading to frequent micronutrient deficiencies in the plants. Incesu et al. (2015) investigated the effects of different mycorrhizal species on plant growth in *D. virginiana* rootstock and found that *C. etunicatum* and *G. clarium* spores were significantly more effective.

Considering this information, we inoculated *D. lotus* rootstock with *C. etunicatum* and *G. clarium* mycorrhizal species spores and examined plant growth, chlorophyll content, photosystem II (PSII) efficiency, and root/leaf nutrient concentrations under four different salt concentrations. The aim of this work is to assist in selecting the most suitable mycorrhiza species response and salt levels to persimmon seedlings. The hypothesis of this work states that, under Mediterranean basin soil conditions, mycorrhiza species reduce salt stress effects on persimmon seedlings.

## 2 Materials and methods

### 2.1 Plant material and AMF inoculations with salinity treatments

Seeds of *Diospyros lotus* L. were obtained from Cukurova University, Faculty of Agriculture, Department of Horticulture’s Persimmon Germplasm orchard (37°1′51.26”N, 35°22′4.43”E). The experiment was carried out under greenhouse conditions. Pots were surface-sterilized with ethanol at a concentration of 70% before being filled with the growth media. *D. lotus* L. seeds were surface-sterilized with sodium hypochlorite solution (1% active chlorine) for 10 min, rinsed three times, and then soaked in distilled water several times.

The experiment was performed in an andesitic tuff, soil, and compost (6:3:1 v/v) mixture (Satir et al., 2016). The growth medium was autoclaved at 121°C for 2 h before use. Three-liter container pots were used under greenhouse conditions.

The Menzilat soil series material was collected from surface horizons of clay loam soil (0–20 cm) in Cukurova Basin (South Turkey). Menzilat soil pH is 7.45, with a low organic matter content (1.41%), a high CaCO_3_ content (28%), and 0.5 M NaHCO_3_ (pH 8.5) extractable 4.55 kg day^−1^ phosphorus.

**Figure 1:**
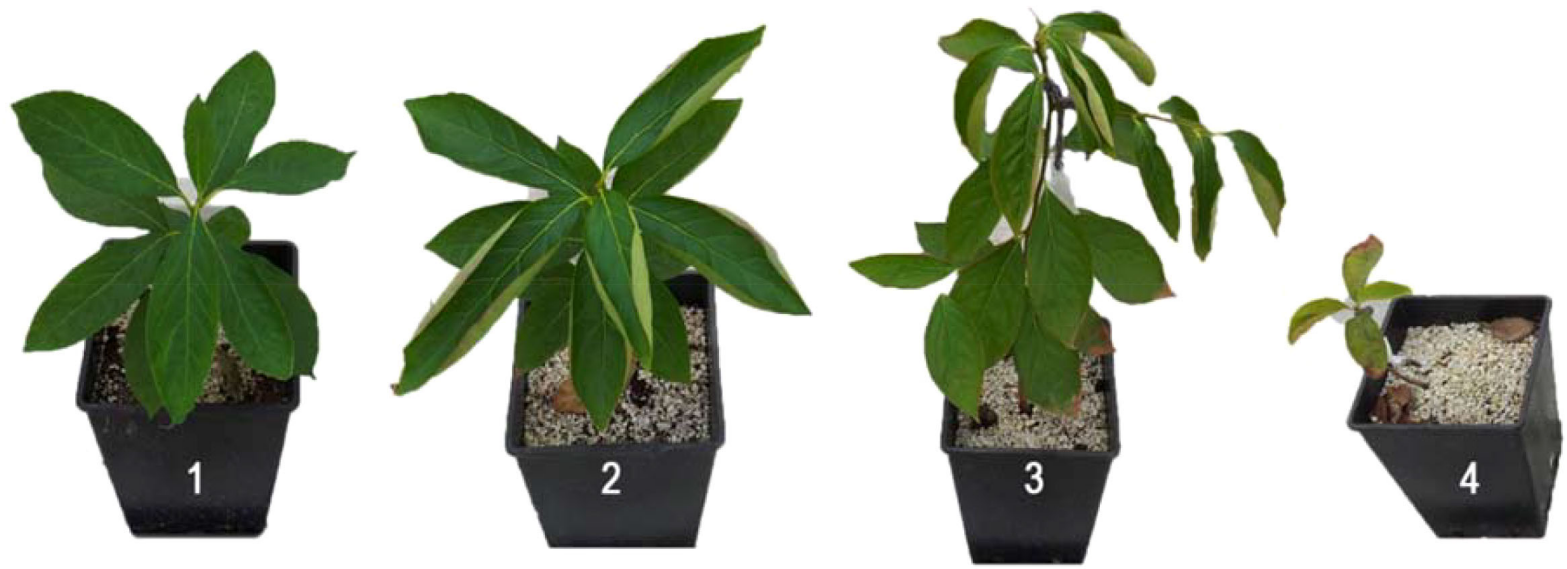
Symptom score ranks: 1—normal healthy green plants without any injury; 2—slightly wilted, damages on leaf tip; 3—moderate to severe damage on leaves (70% of defoliated leaves); 4—most leaves with drying damages (90% of defoliated leaves).

Eight weeks after sowing the seeds of *D. lotus*, inoculated and non-inoculated uniform seedlings at three to four true-leaf stages with the AMF species were transplanted into 3-L plastic pots. A total of 1,000 spores from each of the mycorrhizae species *C. etunicatum* (Becker & Gerdemann) and *G. clarium* (Nicolson & Schenck) were applied 3 cm below each seedling root. For the mycorrhizal inoculum, *G. clarium* and *C. etunicatum* Rothamstedt’s UK isolate were used. A thousand spores were planted 30 mm beneath the seedling bed. An equal quantity of sterilized non-mycorrhizal inoculum was added to the control pots (non-inoculated). The dried leaves were ground, ashed, and then dissolved in an ash solution. The same amount of growth medium without mycorrhiza was added to the non-inoculated plants. Mycorrhizal and non-mycorrhizal seedlings were grown under greenhouse conditions. The pots were periodically and manually watered to keep the soil moisture at field capacity. The plants were grown in a greenhouse at 30–35°C and a relative humidity of 70%–85%, with a 16-h light and an 8-h dark photoperiod. After inoculation of the plants with AMF, the seedlings were treated with four different salinity levels (0, 50, 100, and 150 mM NaCl) when the plants were 6 months old. Seedlings were progressively adapted to salt stress to avoid osmotic shock. Stress conditions were maintained for 70 days. During this period, plants were irrigated with 500 mL of water three times a week. At the end of the experiment, the severity of leaf symptom score was ranked as follows (**Figure 1**): 1—normal healthy green plants without any injury; 2—slightly wilted, damages on leaf tip; 3—moderate to severe damage on leaves (70% of defoliated leaves); and 4—most leaves with drying damages (90% of defoliated leaves).

### 2.2 Recording observations

#### 2.2.1 Leaf chlorophyll concentration and fluorescence measurements

The concentrations of chlorophyll per area were estimated in the same attached leaves as those for which gas exchange measurements were taken using a SPAD portable apparatus (Minolta Co., Osaka, Japan). The chlorophyll fluorescence parameter (*Fv*′/*Fm*′) was measured with a portable fluorimeter (Photon System Instruments Ltd). Readings were recorded at three mature leaves located in the mid-stem zone of each plant at the end of the experiment at mid-day (Gil-Muñoz et al., 2018).

#### 2.2.2 Growth parameters and root colonization

After SPAD, and chlorophyll fluorescence measurements, the plants were harvested, and leaf number and stem length per plant were measured. Furthermore, at the end of the experiment, plants were removed from the pots and separated into roots and shoots, then washed with deionized water. Plant tissues were cleaned and dried at 72°C until the weights were stabilized using a thermos-ventilated oven, then the dry weights of shoot and root were recorded. Collected roots were separated from the growth medium by washing in running tap water and then with distilled water. Roots were dried on tissue paper. Before drying, small sub-samples were taken from roots and preserved in a mixture of ethanol, glacial acetic acid, and formalin for determination of root length and mycorrhizal infection levels. Roots were stained as described by Koske and Gemma (1989) and determined by the method of Giovannetti and Mosse (1980).

#### 2.2.3 Mycorrhizal dependency

Mycorrhizal dependency (MD) was determined by Plenchette et al. (1983), with an equation expressing the difference between the dry weight of the mycorrhizal plant and the dry weight of the non-mycorrhizal plant as a percentage of the dry weight of the mycorrhizal plant:


$$\text{MD}=\frac{\text{DW Mycorrhizal Plant}–\text{DW Non Mycorrhizal Plant}}{\text{DW Mycorrhizal Plant}}\times 100$$

#### 2.2.4 Leaf and root mineral analysis

Dried root and shoot materials were ground. Chloride concentration was determined by using a scientific chloride analyzer. K, Ca, Zn, and Na were determined by atomic absorption spectrophotometry. P concentration was analyzed with a spectrophotometer (Murphy and Riley, 1962).

### 2.3 Experimental design and data analysis

The experiment was arranged as 3 × 4 × 10, i.e., 3 AMF treatments, 4 salinity levels, and 10 replicates, respectively, in a “complete randomized design”. Data were subjected to two-way analysis of variance (ANOVA). AMF root colonization percentage was arc sin transformed for ANOVA. Means of replicates were compared by using the LSD test at *p* ≤ 0.05. All statistical analyses were subjected to SAS v9.00 software. In addition, the correlation coefficients between all measured parameters were calculated according to Pearson’s method. RStudio statistics software was used for data visualization.

## 3 Results

### 3.1 Root colonization (%) and growth parameters

Plant root colonization varied by mycorrhizal inoculation from 18.0% to 59.0% in terms of salinity treatments (**Table 1**). Control treatment had no root colonization because the growth medium was sterilized, I and mycorrhiza, salinity level treatments, and their interaction were statistically significant (*p* ≤ 0.01). For the mycorrhiza treatment, the highest root colonization was obtained in *C. etunicatum* treatment (48.92%). Root colonization of *G. clarium* was found to be 33.0% (**Table 1**). In general, increasing salt levels decreased root colonization.

**Table 1:** Root colonization of *D. lotus* seedlings inoculated with different mycorrhizal species under different salinity levels.

Mycorrhizal species	NaCl	Root colonization (%)
*G. clarium*		33 b
*C. etunicatum*		49 a
Control		0 c
	0 mM	37 a
	50 mM	30 b
	100 mM	26 c
	150 mM	16 d
Control	0 mM	0 f
	50 mM	0 f
	100 mM	0 f
	150 mM	0 f
*G. clarium*	0 mM	51 b
	50 mM	36 c
	100 mM	27 d
	150 mM	18 e
*C. etunicatum*	0 mM	59 a
	50 mM	56 ab
	100 mM	50 b
	150 mM	31 cd
*Prob > F*_(AMF)_		<0.0001*
*Prob > F*_(NaCl level)_		<0.0001*
*Prob > F*_(AMF*NaCl)_		<0.0001*
*LSD*_(AMF) 0.05_		3.543
*LSD*_(NaCl level) 0.05_		4.099
*LSD*_(AMF*NaCl) 0.05_		7.086

*statistically significant according to a two way anova.

Results showed that the main effect of mycorrhiza and salt application and their interaction with fresh weight were statistically significant. In mycorrhiza treatment, the highest fresh weight was determined in plants inoculated with *C. etunicatum* (28.87* g*) and *G. clarium* (24.21 g), whereas the lowest fresh weight was found in control plants (2.92 g) (**Table 2**).

**Table 2:** Fresh weight (g), dry weight (g), leaf Cl concentration (SPAD readings), chlorophyll fluorescence (*Fv*′/*Fm*′), and symptom score of AMF inoculated *D. lotus* under salinity stress.

Species	NaCl	Fresh weight (g)	Dry weight (g)	SPAD	*Fv*′/*Fm*′	Symptom score^*^
*G. clarium*		24.21 a	6.92 a	38.10 a	0.6653 a	1.88 b
*C. etunicatum*		28.87 a	8.07 a	38.55 a	0.7046 a	1.58 c
Control		2.92 b	1.30 b	25.06 b	0.2692 b	2.33 a
	0 mM	26,60	7,81	34.81	0.6172 a	1.00 c
	50 mM	23,48	6,26	35.06	0.5951 a	1.39 b
	100 mM	14,34	4,42	33.69	0.5168 b	2.67 a
	150 mM	10,24	3,22	32.05	0.4563 c	2.67 a
*G. clarium*	0 mM	32.07 ab	8.46 b	40.17	0.7350 a	1.00 d
	50 mM	25.06 bc	7.23 bc	40.20	0.7279 a	1.17 d
	100 mM	23.62 bc	7.45 bc	35.02	0.6550 c	2.83 ab
	150 mM	16.08 cd	4.53 cde	37.03	0.5432 c	2.50 bc
*C. etunicatum*	0 mM	42.22 a	12.80 a	39.95	0.7339 a	1.00 d
	50 mM	42.06 a	10.26 ab	37.73	0.7201 ab	1.00 d
	100 mM	17.44 c	4.72 cd	40.17	0.6945abc	2.17 c
	150 mM	13.76 cde	4.50 cdef	36.33	0.6699 bc	2.17 c
Control	0 mM	5.52 def	2.17 defg	24.32	0.3829 d	1.00 d
	50 mM	3.31 ef	1.29 efg	27.25	0.3374 d	2.00 c
	100 mM	1.97 f	1.11 fg	25.88	0.2009 e	3.00 ab
	150 mM	0.89 f	0.64 g	22.78	0.1558 e	3.33 a
*Prob > F*_(AMF)_		<0.0001*	<0.0001*	<0.0001*	<0.0001*	<0.0001*
*Prob > F*_(NaCl level)_		<0.0001*	<0.0001*	0.0337^*^	<0.0001*	<0.0001*
*Prob > F*_(AMF*NaCl)_		0.0076*	0.0218*	0.9615	<0.0001*	0.0464*
*LSD*_(AMF) 0.05_		5.756	1.697	2.757	0.051	0.280
*LSD*_(NaCl level) 0.05_		6.646	1.959	3.184	0.058	0.324
*LSD*_(AMF*NaCl) 0.05_		11.512	3.394	N.S.	N.S.	0.561

^*^Symptom score ranks: 1—normal healthy green plants without any injury; 2—slightly wilted, damages on leaf tip; 3—moderate to severe damage on leaves (70% of defoliated leaves); 4—most leaves with drying damages (90% of defoliated leaves).

The main effect of mycorrhiza species and salt dose application and their interaction statistically significantly increased dry weight. In mycorrhiza treatment, the highest dry weight was determined in plants inoculated with *C. etunicatum* (8.07* g*) and *G. clarium* (6.92 g), whereas the lowest fresh weight was determined in control plants (1.30 g) (**Table 3**). Mycorrhiza treatments were significant on dry weight. Generally, increasing the salt level decreased plant dry weight. The dry weight was the highest in the 0 mM NaCl and 50 mM NaCl treatment (7.81 g and 6.26 g, respectively), and the lowest root weight was determined in the 100 mM NaCl (4.42 g) and 150 mM NaCl treatment (3.22 g).

**Table 3:** Mycorrhizal dependency of *D. lotus* seedlings inoculated with different mycorrhizal species under different salinity levels.

Species	NaCl	MD (%)	Development (%)
Control		–	–
*G. clarium*		83.07	590.61
*C. etunicatum*		74.39	390.48
*G. clarium*	0 mM	–	–
	50 mM	82.20	561.80
	100 mM	85.11	671.78
	150 mM	85.80	704.12
*C. etunicatum*	0 mM	–	–
	50 mM	87.46	797.25
	100 mM	76.49	425.29
	150 mM	85.70	699.35
Control	0 mM	–	–
	50 mM	–	–
	100 mM	–	–
	150 mM	–	–

MD and growth responses were calculated, and both *G. clarium* and *C. etunicatum* showed high MD% under saline conditions. *G. clarium*-treated seedlings had 83.07% and *G. etunicatum-*inoculated seedling had 74.39% MD. *C. etunicatum* showed the highest MD% at 50 mM (87.46%), but it dropped significantly at 100 mM (76.49%) and then increased again at 150 mM (85.70%).

Plant growth of plants inoculated with *G. clarium* has been increased as 590.61%, and it was increased by *G. etunicatum* inoculation 390.48% in **Table 3**. *C. etunicatum* enhanced development more significantly at 50 mM NaCl (797.25%) than *G. clarium* (561.80%). At higher salinity (100 mM), *G. clarium* performed better (671.78%) than *C. etunicatum* (425.29%).

### 3.2 Leaf chlorophyll concentrations, fluorescence, and symptom score

The leaf chlorophyll concentrations indicated the significant main effects of mycorrhiza and salinity treatments (*p* ≤ 0.01), whereas their interaction effect was not significant (**Table 2**). Plants inoculated with *C. etunicatum* (38.55) and *G. clarium* (38.10) treatments had higher chlorophyll concentrations than non-treated plants (25.06). Moreover, different salinity levels significantly affected the leaf chlorophyll concentration of *D. lotus* seedlings. The highest chlorophyll concentration was determined in control (35.86) plants, whereas it was the lowest in seedlings treated with a high level of salt application, 150 mM NaCl (32.05) (**Table 2**).

Chlorophyll fluorescence in the light-adapted stage (*Fv*′/*Fm*′) of *D. lotus* leaves was significantly affected by the main effects of AMF and salinity treatments. PSII efficiency in the leaves of plants inoculated with *C. etunicatum* (0.7046) and *G. clarium* (0.6653) had higher chlorophyll fluorescence than the control seedlings (0.2692). Furthermore, different salinity levels significantly affected the (*Fv*′/*Fm*′) of *D. lotus* leaves, according to the two-way ANOVA conducted (**Table 3**). The highest chlorophyll fluorescence was determined in control plants (0.7350), whereas it was the lowest in the 150 mM (0.4543) NaCl-treated plants (**Table 2**).

AMF treatments significantly affected the leaf salinity symptom scores of *D. lotus*, according to the statistical analysis conducted. *C. etunicatum* and *G. clarium* mycorrhizae species-inoculated seedlings had lower symptom scores in comparison to non-inoculated plants, 1.58 and 1.88, respectively (**Table 2**).

### 3.3 Leaf nutrition elements

Mycorrhizae species inoculation significantly affected the leaf Ca concentration of *D. lotus. G. clarium*-inoculated plants (1.77%) had the highest leaf Ca concentrations in comparison to non-inoculated plants (0.85%) (**Table 4**). In terms of the AMF × salt treatment interaction, the highest leaf Ca concentration was determined in *G. clarium*-inoculated and 0 mM NaCl-treated plants (1.95%) (**Table 4**).

**Table 4:** Leaf Ca (%), Fe (ppm), K (%), Mg (%), Mn (ppm), Na (%), P (%), Zn (ppm), and Cl (mg L^−1^) concentrations of AMF inoculated *D. lotus* under salinity stress.

Species	NaCl	Ca (%)	K (%)	P (%)	Mg (%)	Fe (ppm)	Mn (ppm)	Zn (ppm)	Na (%)	Cl (%)
*G. clarium*		1.77 a	1.11 a	0.14 a	0.47 a	108.73 a	44.80 a	19.28 a	0.17 b	112.54 b
*C. etunicatum*		1.49 b	1.11 a	0.12 b	0.40 b	88.15 b	42.50 a	13.04 b	0.10 c	122.79 a
Control		0.85 c	0.94 b	0.04 c	0.29 c	63.40 c	26.51 b	12.28 b	0.23 a	91.04 c
	0 mM	1.32	0.75 c	0.10	0.40	84.62 ab	39.63	15.93 a	0.08 c	47.89 c
	50 mM	1.41	1.03 b	0.10	0.39	75.56 b	36.86	13.14 b	0.15 b	110.11 b
	100 mM	1.47	1.20 ab	0.11	0.40	95.68 a	39.51	14.02 ab	0.24 a	138.50 a
	150 mM	1.27	1.23 a	0.09	0.37	91.19 a	35.74	16.37 a	0.19 ab	138.67 a
*G. clarium*	0 mM	1.95 a	0.79 de	0.16 a	0.55 a	120.30 a	55.00 a	22.77 a	0.06 d	53.33 f
	50 mM	1.79 ab	1.12 bc	0.14 abc	0.47 ab	94.60 bc	42.73 bc	17.90 bc	0.13 cd	126.50 cd
	100 mM	1.83 ab	1.36 ab	0.15 ab	0.47 ab	129.20 a	49.50 ab	20.50 ab	0.37 a	132.50 bc
	150 mM	1.51 bc	1.16 abc	0.10 d	0.40 bcd	90.83 c	31.97 de	15.93 cd	0.11 d	137.83 bc
*C. etunicatum*	0 mM	1.26 c	0.68 e	0.12 cd	0.33 cde	77.43 cd	35.70 cd	12.57 def	0.09 d	50.83 fg
	50 mM	1.71 ab	1.25 abc	0.13 bcd	0.45 b	79.80 cd	46.83 ab	13.47 def	0.08 d	131.67 bc
	100 mM	1.28 c	1.06 cd	0.10 d	0.39 bcd	81.23 cd	34.33 cd	10.97 efg	0.12 d	166.83 a
	150 mM	1.69 ab	1.43 a	0.14 abc	0.43 bc	114.13 ab	53.13 a	15.17 cde	0.12 d	141.83 b
Control	0 mM	0.74 d	0.77 de	0.02 f	0.31 de	56.13 e	28.20	12.47 def	0.08 d	39.50 g
	50 mM	0.74 d	0.70 e	0.02 f	0.24 e	52.27 e	21.00 f	8.07 g	0.24 b	72.17 e
	100 mM	1.30 c	1.18 abc	0.06 e	0.33 cde	76.60 cd	34.70 cd	10.60 fg	0.23 bc	116.17 d
	150 mM	0.63 d	1.10 bc	0.03 ef	0.27 e	68.60 de	22.13 ef	18.00 bc	0.34 ab	136.33 bc
*Prob > F*_(AMF)_		<0.0001*	0.0444*	<0.0001*	<0.0001*	<0.0001*	<0.0001*	<0.0001*	0.0001*	<0.0001*
*Prob > F*_(NaCl level)_		0.1604	<0.0001*	0.2122	0.7057	0.0063*	0.4351	0.0372*	<0.0001*	<0.0001*
*Prob > F*_(AMF*NaCl)_		<0.0001*	0.0142*	0.0004*	0.0105*	0.0001*	<0.0001*	0.0004*	0.0002*	<0.0001*
*LSD*_(AMF) 0.05_		0.163	0.153	0.016	0.049	10.063	4.995	2.168	0.055	6.854
*LSD*_(NaCl level) 0.05_		NS	0.177	NS	NS	NS	0.063	1.598	11.619	7.914
*LSD*_(AMF*NaCl) 0.05_		0.325	0.306	0.032	0.098	9.909	0.109	4.337	20.125	13.708

*statistically significant according to a two way anova.

Results of leaf K concentrations indicated that both main and interaction effects were significant on *D. lotus* (**Table 4**). Regarding AMF treatments, the highest leaf K concentration was determined in *C. etunicatum* and *G. clarium* (1.11%); the lowest K concentration was determined in the leaves inoculated with control plants (0.94%). In terms of the interaction effect, the highest K concentration was obtained from the leaves of *G. etunicatum* with 150 mM NaCl treatment (1.43%), whereas it was the lowest in the leaves of the control and 50 mM NaCl (0.70%)-treated plants.

The highest leaf Mg concentration was determined in the *C. etunicatum* (0.47%)- and *G. clarium* (0.40%)-inoculated plants, and the lowest Mg concentration was determined in the control plant (0.29%). In terms of the AMF × salt treatment interaction, the highest leaf Mg concentration was determined in the *G. clarium*-inoculated and 0 mM NaCl-treated plants (0.55%), whereas the lowest leaf Mg concentration was determined in the control 50 mM NaCl (0.24%)- and control 150 mM NaCl (0.27%)-treated plants (**Table 4**).

AMF treatments significantly affected the leaf P concentration of *D. lotus*, and *G. clarium*-inoculated plants (0.14%) had the highest leaf P concentrations in comparison to non-inoculated plants (0.04%) (**Table 4**). In terms of the AMF × salt treatment interaction, the highest leaf P concentration was determined in *G. clarium*-inoculated and 0 mM NaCl-treated plants (0.16%), whereas the lowest leaf P concentration was determined in control 50 mM NaCl (0.02%)- and control 0 mM NaCl (0.02%)-treated plants (**Table 4**).

In terms of leaf Fe concentration, both the main and the interaction effects were statistically significant. The highest leaf Fe concentration was determined as 108.73 parts per million (ppm) in *G. clarium*, whereas it was the lowest in control plants (63.40 ppm).

Statistically, AMF treatments significantly affected the leaf Mn concentration of *D. lotus. G. clarium* (44.80 ppm)- and *G. etunicatum* (42.50 ppm)-inoculated plants had the highest leaf Mn concentrations in comparison to non-inoculated plants (26.51 ppm).

The highest leaf Zn concentration was determined as 19.27 ppm in *G. clarium*, whereas it was the lowest in control (18.90 ppm) and *C. etunicatum* (13.04 ppm). Regarding the main effect of salinity, leaf Zn concentration was the highest in control plants (15.93 ppm) and 75 mM NaCl-treated plants (16.36 ppm) and the lowest in 50 mM NaCl-treated plants (13.14 ppm). According to the interaction effect, the highest leaf Zn concentration was determined in *G. clarium* and 0 mM NaCl-treated plants (22.77 ppm), whereas the lowest leaf Zn concentration was determined in control and 50 mM NaCl-treated plants (8.07 ppm).

Na concentration of the leaves of *D. lotus* was significantly affected by the AMF and salinity treatments. Based on the main effect of AMF treatments, the highest leaf Na concentration was determined in control plants (0.25%), whereas it was determined as 0.17% in *G. clarium*-inoculated plants and 0.10% in *C. etunicatum-*inoculated ones. In terms of salinity treatment, the highest leaf Na concentration was determined in 150 mM NaCl-treated plants (0.28%) and the lowest was determined in control plants (0.07%). In terms of the AMF × salt treatment interaction, the highest leaf Na concentration was determined as 0.37% in the 150 mM NaCl application of *G. clarium*-treated plants.

Both main effects and their interactions statistically significantly affected the leaf Cl concentration of *D. lotus*. The control plants without mycorrhiza treatment had a lower leaf Cl concentration (91.04 ppm) than *C. etunicatum* and *G. clarium*, 122.79 and 112.54 ppm, respectively (**Table 4**). In terms of salinity treatment, the lowest leaf Cl concentration was determined in the leaves of plants treated with 0 mM (47.89 ppm) NaCl. Generally, leaves accumulated more Cl ions with the increasing NaCl doses. In terms of the AMF × salt treatment interaction, the lowest leaf Cl concentration in 0 mM NaCl treatment was determined in the leaves of non-inoculated plants (39.50 ppm). *G. clarium-*treated seedlings accumulated 126.50, 132.50, and 137.83 ppm Cl in 50, 75, and 100 mM NaCl, respectively (**Table 4**). On the other hand, plants inoculated with *G. etunicatum* accumulated 131.67, 141.83, and 166.83 ppm Cl, respectively. Non-inoculated and 150 mM NaCl-treated seedlings accumulated 143.29 ppm Cl. Leaves of plants inoculated with *C. etunicatum* and treated with 150 mM NaCl accumulated 136.33 ppm Cl concentration.

### 3.4 Root tissue mineral nutrition elements

The main effect of AMF and salinity treatment significantly affected the root Ca concentrations (*p* ≤ 0.01) (**Table 5**). The root seedling K concentration of AMF and AMF × salinity interaction effects were significant. In terms of the AMF × salt treatment interaction effect, the highest root K concentration was determined in *C. etunicatum-*inoculated and 0 mM NaCl (0.66%)-, control 50 mM NaCl (0.67%)-, control 100 mM NaCl (0.65%)-, and control 0 mM NaCl (0.65%)-treated plants, whereas it was the lowest in *G.* etunicatum- and 100 mM NaCl (0.28%)-treated samples. The highest root Mg concentration was determined in *G. clarium*-inoculated seedlings (0.51%) and *C. etunicatum* (0.44%)-inoculated plants, and the lowest Mg concentration was determined in control plants (0.37%). In terms of the AMF × salt treatment interaction, the highest root Mg concentration was determined in control 100 mM NaCl-treated plants (0.64%) whereas the lowest leaf Mg concentration was determined in control 150 mM NaCl (0.23%)-treated plants.

**Table 5:** Root Ca (%), Fe (ppm), K (%), Mg (%), Mn (ppm), Na (%), P (%), Zn (ppm), and Cl (mg L^−1^) concentrations of AMF inoculated *D. lotus* under salinity stress.

Species	NaCl	Ca (%)	K (%)	P (%)	Mg (%)	Fe (ppm)	Mn (ppm)	Zn (ppm)	Na (%)	Cl (%)
*G. clarium*		0.56 b	0.42 b	0.15 a	0.51 a	145.23 b	23.63	57.09 a	0.62 a	69.25 a
*C. etunicatum*		0.50 c	0.46 b	0.11 a	0.44 a	176.52 a	21.45	43.47 b	0.54 a	70.25 a
Control		0.77 a	0.62 a	0.05 b	0.37 b	122.88 b	20.58	27.19 c	0.29 b	44.58 b
	0 mM	0.68 a	0.58 a	0.10	0.45 ab	160.00 ab	25.47	40.54 b	0.18 c	31.56 c
	50 mM	0.61 b	0.46 b	0.12	0.39 b	132.02 bc	21.70	36.76 b	0.44 b	60.50 b
	100 mM	0.61 b	0.49 ab	0.11	0.51 a	172.14 a	20.79	40.39 b	0.69 a	76.61 a
	150 mM	0.55 b	0.47 b	0.08	0.41 b	126.20 c	19.58	52.64 a	0.61 a	76.78 a
*G. clarium*	0 mM	0.59	0.43 bc	0.12	0.53 abc	166.40 b	31.73	49.27 bc	0.22 d	32.67 ef
	50 mM	0.50	0.29 c	0.22	0.46 bc	82.77 cd	21.10	48.83 bc	0.55 c	77.67 bcd
	100 mM	0.59	0.54 ab	0.13	0.49 bc	175.13 b	20.53	56.90 ab	0.79 ab	78.33 bcd
	150 mM	0.57	0.44 bc	0.12	0.56 ab	156.60 b	21.13	73.37 a	0.91 a	88.33 ab
*C. etunicatum*	0 mM	0.57	0.66 a	0.15	0.52 a-c	160.20 b	22.23	38.17 c	0.20 d	27.00 f
	50 mM	0.50	0.43 bc	0.11	0.42 cd	268.84 a	27.33	44.33 bc	0.55 c	70.83 cd
	100 mM	0.50	0.28 c	0.09	0.40 cd	162.03 b	19.10	43.93 bc	0.74 a-c	85.00 a-c
	150 mM	0.44	0.46 bc	0.10	0.43 b-d	130.40 bc	17.13	47.43 bc	0.67 bc	98.17 a
Control	0 mM	0.89	0.65 a	0.04	0.30 de	153.40 b	22.43	34.20 cd	0.13 d	35.00 ef
	50 mM	0.82	0.67 a	0.03	0.29 de	67.27 d	16.67	17.10 e	0.22 d	33.00 ef
	100 mM	0.73	0.65 a	0.11	0.64 a	179.27 b	22.73	20.33 de	0.55 c	66.50 d
	150 mM	0.64	0.52 ab	0.03	0.23 e	91.60 cd	20.47	37.13c	0.25 d	43.83 e
*Prob > F*_(AMF)_		<0.0001*	<0.0001*	0.0010*	0.0006*	<0.0001*	0.3684	<0.0001*	<0.0001*	<0.0001*
*Prob > F*_(NaCl level)_		0.0065*	0.1234	0.6048	0.0185*	0.0103*	0.1243	0.0102*	<0.0001*	<0.0001*
*Prob > F*_(AMF*NaCl)_		0.1046	0.0027*	0.1691	<0.0001*	<0.0001*	0.0905	0.0214*	0.0426*	<0.0001*
*LSD*_(AMF) 0.05_		0.062	0.091	0.049	0.068	25.355	NS	8.378	0.116	8.228
*LSD*_(NaCl level) 0.05_		0.071	NS	NS	0.079	29.397	NS	9.674	0.134	9.501
*LSD*_(AMF*NaCl) 0.05_		NS	0.182	NS	0.137	50.089	NS	0.0214	0.0258	16.456

*statistically significant according to a two way anova.

AMF treatments significantly affected the root P concentration of *D. lotus*, according to the two-way ANOVA conducted. *G. clarium* (0.15%)- and *G. etunicatum* (0.11%)-inoculated plants had the highest leaf P concentrations in comparison to non-inoculated plants (0.05%) (**Table 5**). Salinity levels and the AMF × salt treatment interaction were not significant on leaf P concentration. Root P concentration varied by 0.03% to 0.22% in terms of the AMF × salt treatment interaction.

AMF, salinity, and their interaction effects were statistically significant on root Fe concentrations and in mycorrhiza treatments, and the highest root Fe concentration was determined as 180.37 ppm in *G. etunicatum-*inoculated plants, whereas it was the lowest in control plants (122.88 ppm). AMF, salinity, and their interaction effects were not statistically significant on root Mn concentrations. Root Mn concentrations varied by mycorrhizal inoculation from 16.67 to 31.73 ppm in the AMF × salt treatment interaction. In mycorrhiza treatments, the highest root Zn concentration was determined as 57.09 ppm in *G. clarium*-inoculated plants, whereas it was the lowest in control plants (27.19 ppm). Considering the AMF × salt treatment interaction, the highest root Zn concentration was determined in *G. clarium*-inoculated and 150 mM NaCl (73.37 ppm)-treated plants, and the lowest root Zn concentration was determined in 150 mM NaCl (37.13 ppm)-treated plants without mycorrhiza treatment followed by *G. etunicatum-*inoculated and 50 mM NaCl (38.17 ppm)-treated plants.

In terms of mycorrhiza treatment, the highest root Na concentration was determined in *G. clarium* (0.61%)- and *G. etunicatum* (0.54%)-inoculated *D. lotus* seedlings, whereas it was determined as 0.29% in control plants. Regarding salinity treatment, the highest root Na concentration was determined in 150 mM NaCl-treated plants (0.73%) and the lowest was determined in control plants (0.18%). The highest root Na concentration was determined as 0.91% in *G. clarium*-inoculated and 150 mM NaCl-treated plants (**Table 5**). The lowest root Na concentration was determined in *G. clarium*-inoculated and 0 mM NaCl (0.22 ppm)-treated plants together with *G. etunicatum-*inoculated and 0 mM NaCl (0.20 ppm)-, control 50 mM NaCl (0.22 ppm)-, control 100 mM NaCl (0.25 ppm)-, and control 50 mM NaCl (0.13 ppm)-treated plants.

Both main effects and their interactions statistically significantly affected the root Cl concentration of *D. lotus* (**Table 5**). Root Cl concentration was higher in *C. etunicatum*-inoculated seedlings (70.25 ppm) in comparison to control plants (44.58 ppm). Comparing the main effects of salt treatments, the highest root Cl concentrations were found in 100 mM (76.61 ppm) and 150 mM (76.78 ppm) NaCl treatment, whereas it was the lowest in control (31.55 ppm). In terms of the AMF × salt treatment interaction, the highest root Cl concentration was detected in *C. etunicatum-*inoculated and 150 mM NaCl (98.17 ppm)-treated plants, whereas it was the lowest in *C. etunicatum-*inoculated 0 mM NaCl (27.00 ppm)-treated plants (**Table 5**).

### 3.5 Correlation coefficient analysis

**Figure 2:**
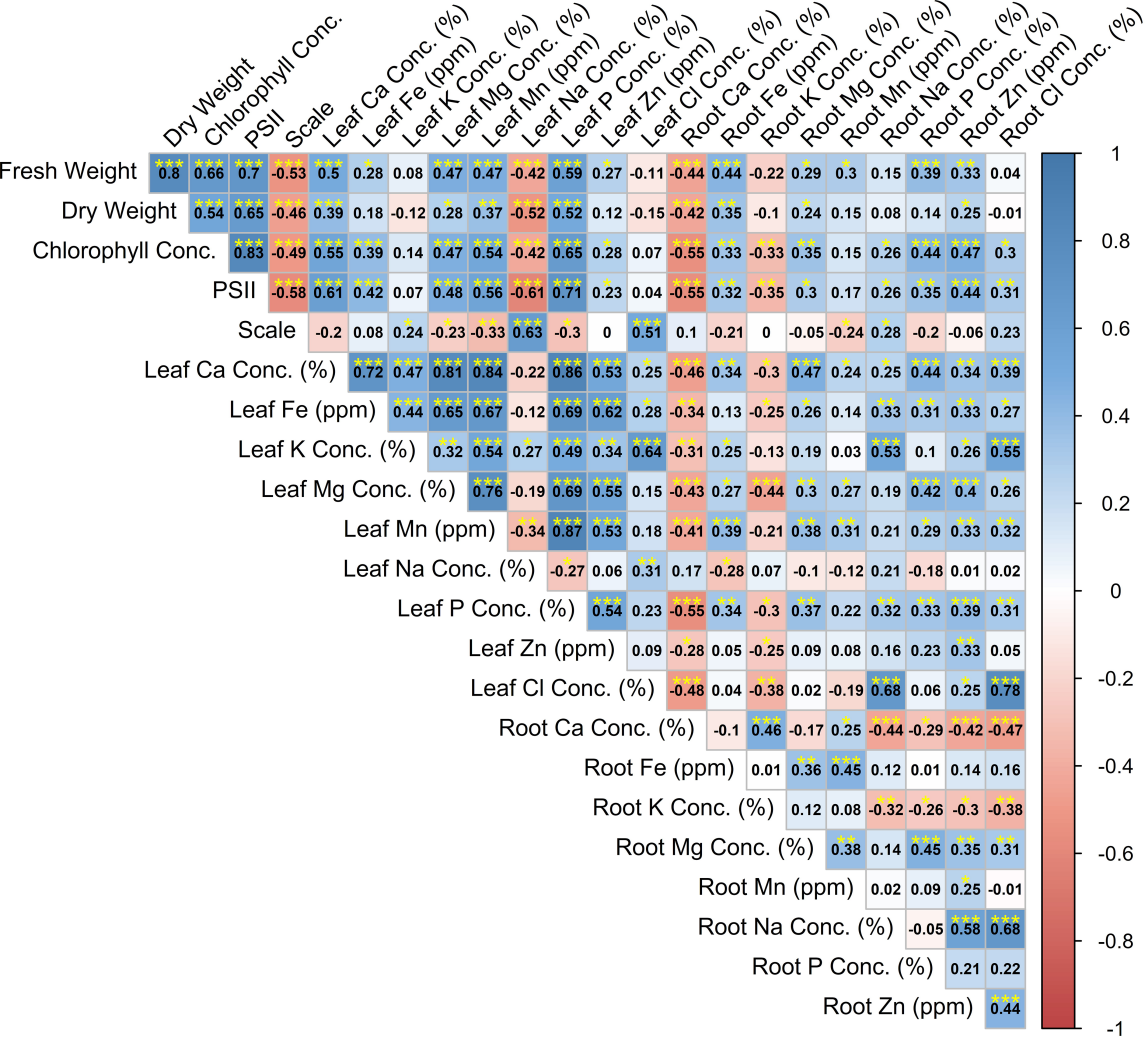
Correlation coefficient analysis between investigated variables.

Significant correlations between investigated parameters were determined (**Figure 2**). The correlation coefficients between symptom score and leaf Na concentration (0.63) and between leaf Na concentration and PSII (−0.61) were significant. Also, significant correlations were determined between dry weight and PSII (0.65), between fresh weight and PSII (0.70), and between fresh weight and SPAD (0.66). The correlation coefficients between PSII and SPAD (0.83), between PSII and leaf P concentration (0.71), between PSII and leaf Mg concentration (0.61), and between SPAD and leaf P concentration (0.65) were significant (**Figure 2**). The correlation coefficients between leaf Mn concentration and leaf P concentration (0.87), between leaf Mn concentration and leaf Mg concentration (0.76), between leaf Mn concentration and leaf Ca concentration (0.84), between leaf Mn concentration and leaf Fe concentration (0.67), between leaf P concentration and leaf Fe concentration (0.69), between leaf Mg concentration and leaf Fe concentration (0.65), between leaf Ca concentration and leaf Fe concentration (0.72), and between leaf Zn concentration and leaf Fe concentration (0.72) were found to be significant. Also, significant correlations were determined between root Cl and root Na (0.68), between root Cl and leaf Cl (0.78), between leaf K and leaf Cl (0.64), and between root Na and leaf Cl (0.68) (**Figure 2**).

**Figure 3:**
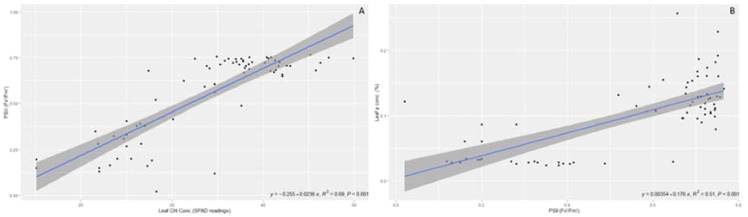
Regressions between leaf chlorophyll concentrations vs. PSII **(A)** and leaf P concentrations vs. PSII **(B)**.

Regression analyses were performed between these correlations in order to predict plant growth based on some of the highly correlated variables under different AMF inoculations. **Figure 3** presents the significant regression between leaf Cl concentration and PSII (*R* ^2^ = 0.69, *p* < 0.001). Regression between leaf P concentration and PSII was also significant (*R^2^ =* 0.51, *p* < 0.001) according to the regression analysis conducted.

## 4 Discussion

The inoculation of mycorrhiza species and salinity levels affected *D. lotus* seedling growth, and some physiological characteristics of mineral nutrient uptake were evaluated. When root colonization was examined, colonization decreased as salt doses increased. AMF can adapt to and survive in a saline environment, but high salt stress may inhibit infection development, prevent hyphal growth, reduce spore viability, decrease spore density, and reduce AMF biomass (Juniper and Abbott, 2006). Additionally, it has been reported that Na^+^ reduces colonization rates by exerting a direct toxic effect on AMF functions (Boorboori and Lackoova, 2024). In *D. lotus* roots, *C. etunicatum* was able to propagate much better than *G. clarium* even under saline conditions. Incesu et al. (2015), in their study examining the effects *of G. mosseae, G. clarium, C. etunicatum, G. caledonium*, and *G. intraradices* on *D. virginiana* rootstocks, found colonization levels of *C. etunicatum* at 36% and *G. clarium* at 28%. Similar to *D. lotus, C. etunicatum* also propagated much more successfully in *D. virginiana* rootstocks. These studies suggest that different mycorrhizal species may be more effective for different plants.

Machineski et al. (2018) investigated the effects of *Dentiscutata heterogama, C. etunicatum, G. clarus, Acaulospora scrobiculata*, and *A. morrowiae* on *D. kaki* seedlings. They found that *D. heterogama* showed 36.67% colonization, while *C. etunicatum* achieved only 16.67%. On the other hand, Ortas et al. (2002) reported in their study on citrus plants that *G. clarium*-colonized rootes were much more affected than *C. etunicatum*. The results we obtained indicate that in *D. lotus*, *C. etunicatum* was more successful in colonization compared to *C. clarium* and could even propagate under saline conditions. Although *G. clarium* did not proliferate as well in the roots as *C. etunicatum*, it still had positive effects on plant growth. Giri and Mukerji (2004) suggested that under salt stress, plants rely on mycorrhiza to adapt and sustain nutrient uptake throughout their growth stages. Although AM colonization often decreases with rising salinity, plants’ dependence on AMF increases. This implies that once the symbiotic relationship was formed, the association between AMF and plants becomes more crucial in saline soil conditions. In this study, although *G. clarium* did not proliferate in the roots as effectively as *C. etunicatum*, its positive effects on plant growth require further detailed investigation. Since the plants in this study were propagated by seed, examining vegetatively propagated sample plants would provide greater clarity on this issue.

**Figure 4:**
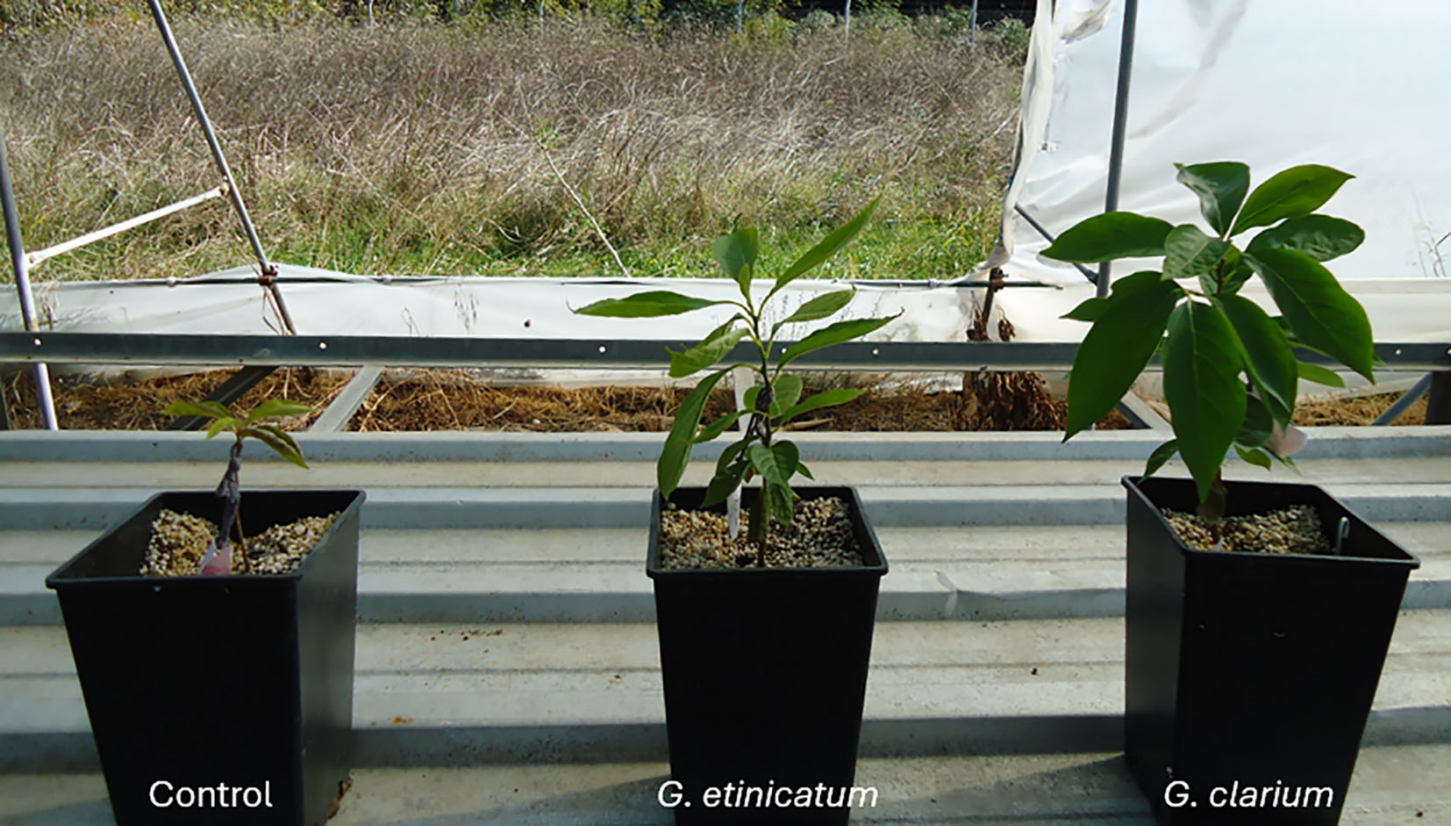
*D. lotus* seedlings treated with AMF.

Plant growth percentage revealed that *D. lotus* plants could not grow without mycorrhiza, as presented in **Figure 4**. This shows that *D. lotus* plants are highly dependent on mycorrhiza. Yan et al., 2023 results show that *D. lotus* is AMF-dependent, which supports the present work. Previously, several studies reported that mycorrhizal inoculation positively affected many plant cultivars’ growth (Waterer and Coltman, 1989; Matsubara and Hosokawa, 1999; Ortas et al., 2002; Oyetunji et al., 2007; Ortas et al., 2011; Wu et al., 2011; Incesu et al., 2015).

When examining fresh and dry weights, it was observed that *C. etunicatum* increased plant growth more than G. *Clarium*. These data indicate that *C. etunicatum* can be used for the cultivation of lotus rootstocks. Better seedling development will also accelerate the readiness for grafting. The capacity of plants to withstand salt stress is typically measured by their biomass yield (da Silva et al., 2008; Ruiz-Lozano et al., 2012). When dry weights were examined, *C. etunicatum* and *G. clarium* were measured at 10.26 and 7.23 g, respectively, under 50 mM NaCl treatment, and 12.80 and 8.46 g, respectively, under 0 mM NaCl (control). These results indicate that in saline soils corresponding to the 50 mM NaCl level, *D. lotus* rootstocks can be inoculated with both mycorrhizae, but *C. etunicatum* proves significantly more effective.

The study was conducted on *C. etunicatum*- and *G. clarium*-inoculated *D. virginiana* seedlings and inoculation increased plant growth to the same extent as the work of Incesu et al. (2015). Qi et al. (2006) reported that *D. lotus* seedlings’ height, number of leaves, stem diameter, leaf area, fresh and dry weights of stem and leaf, and leaf chlorophyll content were significantly improved by AMF species such as *G. mosseae* and *G. intraradices*. There are a limited number of studies on the effects of mycorrhizae in the *Diospyros* genus. These results highlight the need for further research to determine which mycorrhizal species is most effective for each species.

SPAD (chlorophyll content) and PSII efficiency values were found to be lower in non-mycorrhizal plants but higher in plants inoculated with *G. clarium* and *C. etunicatum*. The *Fv*/*Fm* ratio serves as an indicator of the primary photochemical efficiency of PSII, which is highly sensitive to various environmental stressors. Studies, such as that by Sheng et al. (2008), confirm reliability as a stress marker. Salinity stress disrupts the ultrastructure of chloroplasts and other photosynthetic organelles, decreases the photosynthetic pigment content, and impairs the PSII reaction center (Boorboori & Lackoová, 2024). However, plants with salinity tolerance often show improved efficiency when inoculated with AMF (Khalil et al., 2011; Fan et al., 2012; Rodríguez-Morán et al., 2015: Satir et al., 2016). AMF inoculation mitigates the negative impacts of salinity stress on chlorophyll and photosynthetic pigments by detoxifying Na^+^ and preventing its translocation to shoots (Abeer et al., 2014; Chandrasekaran et al., 2019; Khalil et al., 2011). Additionally, AMF enhances chlorophyll synthesis and boosts photosynthetic efficiency by stimulating the activity of chlorophyll synthetase enzymes (Wright et al., 1998; Liang and Shi, 2021).

In the present study, it was observed that lotus plants remained in slightly good conditions in terms of SPAD and PSII despite increasing salt doses. These results indicate that the lotus plant has increased tolerance to salt stress in both *G. clarium* and *C. etunicatum* mycorrhizae infection. Symptom scores showed that the plants exhibited signs of salinity as the dose increased. However, despite this, the plants did not show a decline in SPAD and PSII performance. The most damaging results in symptom scores were observed in non-mycorrhizal plants at 100 mM and 150 mM doses. Lotus plants failed to thrive in sterilized soils, and with increasing salt doses, they displayed significantly lower SPAD and PSII performance. These results confirm that the lotus plant is sensitive to salt. However, it is also necessary to investigate which mycorrhizae are present in the soil where lotus is cultivated. Given that salt tolerance increases in the presence of *G. clarium* and *C. etunicatum*, determining which natural mycorrhizae exist in the growing environment will help assess the extent to which lotus plants are affected by environmental conditions. Shoot and root tissue mineral nutrient analyses were carried out (**Tables 4**, **5**). AMF promote the absorption and transportation of macronutrients and micronutrients such as C, N, P, Fe, Cu, and Zn concentrations in plant tissues. Mycorrhiza species-inoculated *D. lotus* plant tissue have higher Ca, K, Mg, Mn, and Zn than in non-inoculated control seedlings. Our findings are in parallel with the literature (Qi et al., 2006; Shi et al., 2016; Wang and Liu, 2017). The most important element that shows the significant effect of salt on plants is sodium. It is clearly shown that without mycorrhiza inoculation, plant tissue has a higher leaf Na concentration, one of the most important indicator elements of salt stress, and was higher in non-inoculated plant leaves and lower in mycorrhiza-treated plant leaves. It is seen that too much sodium is retained in the AMF-inoculated plant roots (**Table 5**). A similar situation is seen to be valid for elements that are indicators of salinity such as calcium and potassium. The average values of the plants inoculated with mycorrhizal fungi show that the plants take Zn, Mn, Fe, K, Mg, and Cl in their leaves with mycorrhiza inoculation. This means that, in the case of mycorrhizae inoculation, toxic level mineral elements such as Na are kept in the root section, and other non-toxic level elements were transported more to the leaves. The research findings are supported by Marschner (2012) and Ortas and Akpinar (2006).

It has been found that while *G. clarium* was more effective in promoting plant growth, *G. clarium* demonstrated higher performance in leaf nutrient content. Specifically, *G. clarium* was more effective than *C. etunicatum* in the uptake of Ca, Fe, Mg, Na, P, and Zn. Na (sodium), which influences salt tolerance, was found in higher concentrations in *G. clarium*-inoculated plants compared to those inoculated with *C. etunicatum*. This explains why *C. etunicatum*-inoculated plants exhibited greater growth. Although Mg (magnesium), the central molecule of chlorophyll, was higher in *G. clarium*-inoculated plants, Na accumulation negatively affected plant growth. Na levels in *G. clarium*-inoculated plants were 0.37 at 100 mM and 0.11 at 150 mM, a variation that may also be attributed to heterozygosity. In contrast, Cl (chloride) was found in higher concentrations in *C. etunicatum*-inoculated plants.

These results indicate that both mycorrhizae were able to protect the plants from excessive Na accumulation in leaf tissues. However, the same protective effect was not observed for Cl. Lotus plants exhibited high Cl concentrations under both mycorrhizal treatments. According to Evelin et al. (2019), soil salinization reduces phosphorus (P) availability to plants by causing its precipitation with cations like Ca²^+^, Mg²^+^, and Zn²^+^, depending on soil pH. Nevertheless, AMF can enhance P uptake, thereby promoting better growth and development in host plants. In this study, the highest leaf P uptake was obtained in *G. clarium*, followed by *C. etunicatum*. The lowest leaf P concentration was detected in non-mycorrhizal lotus plants. In *C. etunicatum*, leaf P was measured at 0.12% under 0 mM NaCl and at 0.14% under 150 mM NaCl. For *G. clarium*, the highest leaf P content was observed at 0 mM, with a gradual decrease as salt dosage increased; however, it maintained higher P concentrations compared to *C. etunicatum*. Nevertheless, the superior growth performance of *C. etunicatum*-inoculated plants suggests that they utilize absorbed nutrients more efficiently for biomass production. The uptake of essential minerals like Mg decreases due to the antagonistic effect of Na on Mg absorption, which is required for chlorophyll production, leading to lower chlorophyll levels in leaves (Sheng et al., 2008). However, mycorrhizal fungi reduce this Na–Mg antagonism (Giri et al., 2003), enhancing Mg absorption in mycorrhizal plants (Wu et al., 2011). In this study, *G. clarium* and *C. etunicatum* uptake of Mg was higher than in plants without mycorrhiza; thus, there were no dramatic decreases in SPAD and PSII in mycorrhizal-grafted plants. Root Na concentration was found to be the lowest in non-mycorrhizal plants. Leaf Na concentration, on the other hand, was the highest in non-mycorrhizal plants. This indicates that *G. clarium* and *C. etunicatum*, which colonized the roots, did not transport Na to the upper organs. Despite the presence of Na in the environment and its transport to the roots, the low Na concentration in the leaves suggests that the plant has increased salt resistance.

Cl concentration in the roots was found to be twice as high in both mycorrhizal treatments compared to non-mycorrhizal plants. Both root and leaf Cl concentrations were higher in *G. clarium* and *C. etunicatum* applications. This suggests that in lotus cultivation, the Na content in the soil is more critical than Cl. Numerous studies have shown that arbuscular mycorrhizae (AM) enhance plant growth by improving the acquisition of immobile soil nutrients, especially in nutrient-deficient soils. Extensive research documents that AMF colonization boosts the uptake of phosphorus (P), zinc (Zn), copper (Cu), manganese (Mn), and iron (Fe), while also promoting overall plant growth under low-nutrient conditions (Hajiboland, 2013).

The correlation coefficients between leaf Na concentration and PSII were significant. Excessive Na^+^ impairs the uptake of essential nutrients such as K^+^, Ca_2_^+^, P, and N (Rehman et al., 2019), disrupting cellular biochemical, physiological, and molecular functions (Shahid et al., 2020). Photosynthesis plays a crucial role in sustaining plant growth under stress; however, salt stress severely reduces photosynthetic efficiency (Zhang et al., 2018; Ma et al., 1997). Specifically, salinity inhibits the PSII reaction center by impairing the oxygen-evolving complex (OEC) on the donor side and degrading the D1 protein on the acceptor side. This disruption slows electron transfer, causing excess electrons to accumulate in the transport chain. Leaked electrons then react with free oxygen, generating ROS that further damage PSII (Chen et al., 2020; Białasek et al., 2017). Prolonged stress can even trigger thylakoid membrane peroxidation or disintegration (Mitsuya et al., 2000).

Correlation analysis revealed a strong relationship between leaf P and the uptake of Mn and Fe. Studies have shown that mycorrhizae not only enhance P uptake but also increase Zn uptake (Marschner, 1995; Ortas, 2003). Mycorrhizal association significantly improves the uptake of immobile micronutrients, particularly Zn, Cu, and Mn.

When the data were analyzed in the present study, it was determined that the measured data were compatible with mycorrhizal inoculation. In the correlation study, a high correlation was determined between plant development and chlorophyll, PSII, and nutrient elements (**Figure 1**). The research findings provided strong support for the hypothesis that mycorrhizae increase plant development by controlling salt uptake into the plant.

## 5 Conclusion

The current study highlights the beneficial impact of AMF on *D. lotus* seedlings under salinity stress. Both *G. clarium* and *C. etunicatum* significantly enhanced root colonization, photosynthetic efficiency, dry weight, MD, and nutrient concentration compared to non-inoculated control seedlings. Without mycorrhizal inoculation, *D. lotus* plants cannot develop under salt stress conditions. Notably, *C. etunicatum* exhibited superior efficacy under higher salinity levels, maintaining better plant growth, higher chlorophyll fluorescence, and greater mineral nutrient concentration, especially P, Fe, and Zn. Mycorrhizal inoculation substantially reduced symptom severity and moderated ion toxicity by restricting Na and Cl accumulation, thereby maintaining ionic balance critical for plant survival under saline soil conditions. The observed MD (over 80%) under salt stress underscores the importance of AMF symbiosis in improving salinity tolerance in *D. lotus*. The study concludes that inoculating *D. lotus* with *C. etunicatum* could allow its use in the increasingly saline soils of the Mediterranean basin. The results obtained demonstrated that *D. lotus* plants inoculated with *C. etunicatum* can be successfully utilized under 50 mM NaCl salt stress.

## Data Availability

The raw data supporting the conclusions of this article will be made available by the authors, without undue reservation.
